# Treatment and pregnancy outcomes of pregnant women exposed to second-line anti-tuberculosis drugs in South Africa

**DOI:** 10.1186/s12884-021-03956-6

**Published:** 2021-06-28

**Authors:** Idah Mokhele, Nelly Jinga, Rebecca Berhanu, Thandi Dlamini, Lawrence Long, Denise Evans

**Affiliations:** 1 Health Economics and Epidemiology Research Office, Department of Internal Medicine, School of Clinical Medicine, Faculty of Health Sciences, University of the Witwatersrand, Johannesburg, South Africa; 2 Department of Global Health, Boston University School of Public Health, Boston, MA USA; 3 Right To Care, Johannesburg, South Africa

**Keywords:** Multi-drug-resistant tuberculosis, Rifampicin resistance, South Africa, Pregnancy, HIV, Second-line anti-TB treatment

## Abstract

**Background:**

Multi-drug resistant and rifampicin-resistant tuberculosis (MDR/RR-TB) in pregnant women is a cause for concern globally; few data have described the safety of second-line anti-TB medications during pregnancy. We aim to describe TB treatment and pregnancy outcomes among pregnant women receiving second-line anti-tuberculosis treatment for MDR/RR-TB in Johannesburg, South Africa.

**Methods:**

We conducted a retrospective record review of pregnant women (≥ 18 years) who received treatment for MDR/RR-TB between 01/2010–08/2016 at three outpatient treatment sites in Johannesburg, South Africa. Demographic, treatment and pregnancy outcome data were collected from available medical records. Preterm birth (< 37 weeks), and miscarriage were categorized as adverse pregnancy outcomes.

**Results:**

Out of 720 women of child-bearing age who received MDR/RR-TB treatment at the three study sites, 35 (4.4%) pregnancies were identified. Overall, 68.7% (24/35) were HIV infected, 83.3% (20/24) were on antiretroviral therapy (ART). Most women, 88.6% (31/35), were pregnant at the time of MDR/RR-TB diagnosis and four women became pregnant during treatment.

Pregnancy outcomes were available for 20/35 (57.1%) women, which included 15 live births (11 occurred prior to 37 weeks), 1 neonatal death, 1 miscarriage and 3 pregnancy terminations. Overall, 13/20 (65.0%) women with known pregnancy outcomes had an adverse pregnancy outcome. Of the 28 women with known TB treatment outcomes 17 (60.7%) completed treatment successfully (4 were cured and 13 completed treatment), 3 (10.7%) died and 8 (28.6%) were lost-to-follow-up.

**Conclusions:**

Pregnant women with MDR/RR-TB suffer from high rates of adverse pregnancy outcomes and about 60% achieve a successful TB treatment outcome. These vulnerable patients require close monitoring and coordinated obstetric, HIV and TB care.

## Background

Multi-drug resistant and rifampicin-resistant tuberculosis (MDR/RR-TB) remains a significant threat to the global control of tuberculosis (TB). An estimated 465,000 new cases and 182,000 deaths from MDR/RR-TB were reported globally in 2018, with only 56% of cases successfully treated [1, 2]. South Africa ranks among the top ten countries in the world in terms of the number of patients with MDR/RR-TB, which is driven by a high HIV burden [1, 3]. In 2018, South Africa had 13,199 MDR/RR-TB cases detected; 72.4% of these started on treatment and treatment success was similar to the global rate, and mortality was above 20% [1].

An estimated 80% of women initiating second-line anti-TB treatment for MDR/RR-TB in South Africa are of reproductive age (defined as aged 15–44), and 68.0% are also HIV-positive [4]. TB and HIV co-infection in pregnancy are major risk factors for maternal mortality and poor neonatal outcomes [4]. Multiple drugs used in MDR/RR-TB regimens are known to be potentially teratogenic to the foetus, including aminoglycosides and ethionamide [5]. Also, there is limited data from animal studies that linezolid, fluoroquinolones, clofazimine, terizidone and delamanid could be potentially unsafe in pregnancy. However, there is minimal human data as pregnant/breastfeeding women are routinely excluded from clinical trials [6, 7]. However, the potential risk associated with MDR/RR-TB treatment to the foetus must be weighed against delayed treatment which risks obstetric and neonatal complications, on-going transmission, treatment failure and amplification of resistance from sub-optimal regimens [6–8]. Current South African and World Health Organization (WHO) guidelines for the treatment of MDR/RR-TB recommend initiating standard short and long-course regimens for MDR/RR-TB in pregnant and breastfeeding women with the exclusion of aminoglycosides and ethionamide [9, 10].

During the study period (2010–2016), the standard of care for MDR/RR-TB treatment in South Africa was a regimen of five drugs (kanamycin, moxifloxacin, ethionamide, terizidone, and pyrazinamide) for six months (intensive phase) followed by 18 months (continuation phase) of four drugs (oral moxifloxacin, ethionamide, terizidone, and pyrazinamide). For patients with documented resistance or intolerance to the drugs in the standard regimen (e.g. pre-extensively drug-resistant TB (preXDR) or extensively drug-resistant TB (XDR-TB)), an individualized regimen containing linezolid, para-aminosalicylic acid, clofazimine, and/or capreomycin was used [11, 12]. In September 2018, the South Africa National TB program eliminated injectables and recommended the use of all-oral regimens, including for pregnant women [13, 14]. However, it will be some time before TB treatment, and pregnancy outcomes for women treated with these new regimens are available given the relatively low incidence of pregnancy during MDR/RR-TB treatment. Meanwhile, data on TB treatment and pregnancy outcomes for women on older long course MDR/RR-TB regimens are still extremely valuable for patients and clinicians as both weigh the safety of second-line TB medications during pregnancy.

The aim of the study is to describe TB treatment and pregnancy outcomes among pregnant women receiving second-line anti-tuberculosis treatment for MDR/RR-TB in Johannesburg, South Africa.

## Methods

### MDR/RR-TB description

MDR-TB is TB that is resistant to at least isoniazid (INH) and rifampicin (RIF), the two most important anti-TB drugs in the first-line treatment regimen [15, 16]. TB that is resistant to RIF but with unidentified or awaiting sensitivities to additional drugs is referred to as rifampicin-resistant TB (RR-TB). MDR-TB with additional resistance to second-line drugs from the fluoroquinolone and injectable drug classes is defined as extensively drug-resistant TB (XDR-TB), while preXDR-TB is MDR-TB which also has resistance to either a fluoroquinolone or a second-line injectable drug.

### Study setting and design

**Fig. 1:**
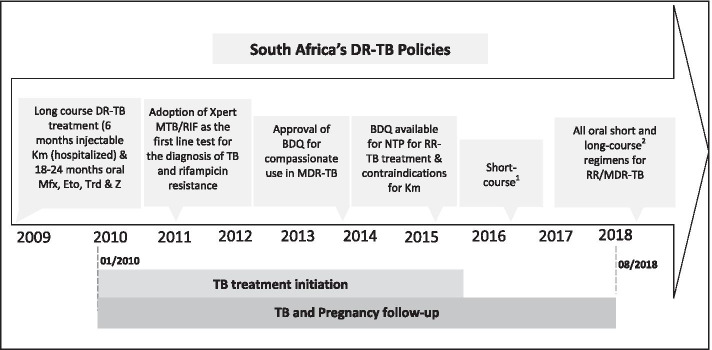
Study period alongside South Africa’s drug-resistant TB policies. DR-TB – drug-resistant tuberculosis, Km – Kanamycin, Mfx – Moxifloxacin, Eto – Ethambutol, INH – Isoniazid, Cfz – Clofazimine, Cfx – Cefozitin, BDQ – Bedaquiline, Trd –Terizidone, Z – Pyrazinamide, NTP- National TB programme, Xpert MTB/RIF – a nucleic acid amplification-based diagnostic system that detects Mycobacterium tuberculosis and rifampin (RIF) resistance in under2 hours. ^1^ Short course: BDQ, LZD, LFX, CFZ, Hi-INH, PZA, EMB × 9–11 months. ^2^ Long course: BDQ, LFX, LZD, TRD and CFZ for 18 months

We conducted a retrospective record review of adult (18–49 years old) women diagnosed with laboratory-confirmed MDR/RR-TB, who initiated second-line anti-TB treatment (defined as a regimen containing at least two second-line agents, including at least one of a fluoroquinolone or second-line injectable agent), between 01/2010–08/2016 at three public outpatient treatment sites in Johannesburg, South Africa and had a pregnancy overlap with their TB treatment (Fig. 1). Two of the three sites are decentralized drug-resistant TB treatment sites and the third the only specialized hospital for the management of MDR-TB and XDR-TB cases in the Gauteng Province. Eligible participants were identified through each site’s respective electronic data management system and MDR/RR-TB registers.

### Data collection

Clinical data on all eligible women were collected from medical records at treatment sites. This included medical, obstetric, drug-exposure histories, treatments and laboratory data for acute and chronic conditions were collected from medical records at treatment sites. Medical records were defined as all electronic or paper documentation of the patient’s medical care at the treatment facilities, including National Health Laboratory Services (NHLS) laboratory reports, prescriptions, MDR/RR-TB patient card, MDR/RR-TB clinic card, antenatal care (ANC) and delivery records and each site’s respective electronic data management system, hospital admission records, and maternal or neonatal death records where applicable.

### Study variables

We collected the following patient sociodemographic characteristics at treatment initiation; age (18–29, ≥ 30 years), nationality (South African or non-South Africa), marital status (in a relationship/married or single, not in a relationship), highest education level (< grade 12 versus ≥ grade 12) and employment status (employed or unemployed). MDR/RR-TB related information collected include the DR-TB treatment site type (centralized or decentralized), year of MDR/RR-TB treatment initiation (2010–2011, 2012–2013 or 2014–2016), MDR/RR-TB treatment regimen and MDR/RR-TB treatment regimen changes during the course of treatment. Additionally, we collected TB drug-resistance profile (RR-TB, MDR-TB, pre-XDR-TB, XDR-TB), patient category (new, previously treated), and classification of disease (pulmonary, extra-pulmonary, pulmonary and extra-pulmonary).

We categorized pregnancy onset as before or after MDR/RR-TB treatment initiation. Among those pregnant after the MDR/RR-TB treatment initiation, we calculated duration on second-line anti-TB treatment as the time (in days) from the start of MDR/RR-TB treatment to the self-reported estimated date of pregnancy onset.

We categorized participants’ HIV status and antiretroviral therapy (ART) status collected from medical records as (HIV-negative, HIV-positive), (on ART, not on ART, ART status unknown) respectively, and collected ART regimens for HIV-positive participants initiated on ART. Additionally, ART initiation was categorized as before or after MDR/RR-TB treatment initiation depending on the timing of ART initiation.

#### Maternal Adverse events

Adverse events during MDR/RR-TB treatment were determined from laboratory results, patient self-report or clinician documentation of adverse events on patient medical records. Loss of weight, dizziness, rash, nausea and ototoxicity and the severity grade were identified and classified as documented by the clinician. The severity of self-reported AEs was graded by clinicians using the Division of AIDS (DAIDS) adverse event’ categorization as mild (grade 1), moderate (grade 2), severe (grade 3), potentially life-threatening (grade 4), based on interference with usual functional, social, and self-care activities as detailed below [17].

- **Mild**: symptoms do not limit daily usual social and functional activities and usually don’t require any medical intervention;
- **Moderate**: symptoms cause greater than minimal interference with usual social and functional activities and require minimal medical intervention;
- **Severe**: symptoms cause inability to perform usual social and functional activities medical interventions including possible hospitalization required;
- **Life threatening:** symptoms cause inability to perform basic self-care functions intervention including hospitalization are required to prevent death or disability.

Nephrotoxicity, hepatotoxicity, anaemia, hypokalaemia and neutropenia were confirmed by monitoring laboratory tests including renal function test, liver function test, haemoglobin test, serum potassium test and absolute neutrophil count, respectively. Adverse events confirmed by laboratory results were graded using the DAIDS adverse event’ categorization as either as mild, moderate, severe or life-threatening [17, 18].

#### Maternal MDR/RR-TB treatment outcomes

MDR/RR-TB outcomes were defined using standard TB outcomes as defined in the WHO definitions and reporting framework for TB as cured, completed, died, failed, lost to follow-up (LTFU), or not evaluated [19].

#### Pregnancy outcomes

Pregnancy outcomes were assigned in patient medical records according to the standard categories as live birth, miscarriage, stillbirth, and termination of pregnancy [20, 21]. Preterm birth (< 37-weeks’ gestation), stillbirth, and miscarriage were categorized as adverse pregnancy outcomes. Women were referred to other facilities for antenatal care and delivery but we did not have access to these records. We relied on documentation of antenatal, delivery and neonatal outcomes in the TB patient record. There is no infant outcome classification proposed as infant outcomes were not available in the records.

### Analysis

We used descriptive statistics to summarize demographic, clinical characteristics, pregnancy and TB treatment outcomes. We describe the frequency and severity of adverse events occurring during MDR/RR-TB treatment. Continuous variables were described using medians and interquartile ranges (IQR) where appropriate. Categorical variables are described using frequencies and percentages.

Differences by HIV status were determined using the Chi-square or Fisher’s exact tests and continuous variables by t-test or Wilcoxon sign-rank-sum test where appropriate. Statistical significance level was set at the 5% level.

Analysis was conducted using STATA version 14 (Stata Corp, College Station, Texas USA).

## Results

### Sample selection

**Fig. 2:**
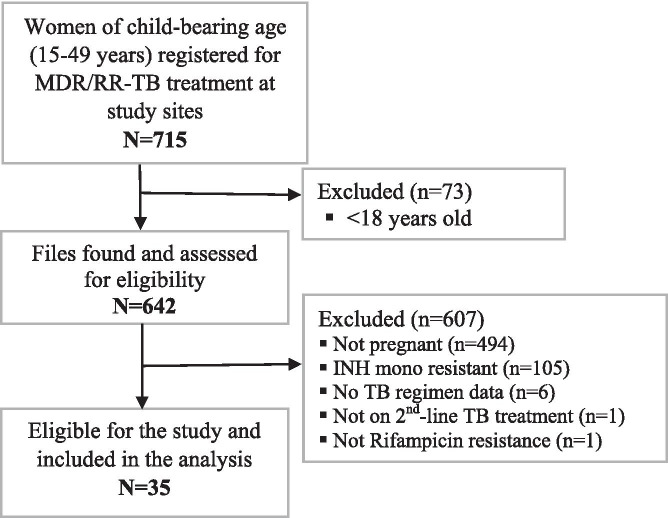
Summary of participant selection

Figure 2 provides a summary of the screening process for sample selection. Of the 715 women aged (15–49 years) initiated on treatment for MDR/RR-TB at the study sites, 35 women (5.0%) were included in the study. We describe the characteristics and outcomes of these 35 women.

### Maternal demographic and TB related characteristics

Almost two-thirds of study participants were treated at a decentralized DR-TB treatment sites. The median age at MDR/RR-TB treatment initiation was 30 years (IQR:25.0–35.0) (Table 1). A total of 19/35 (54.3%) of study participants were not in a relationship. Overall, 8/35 (22.9%) of the women completed high school; however, only a quarter (25.7%) were employed.

**Table 1:** Maternal TB and pregnancy characteristics at start of TB treatment by HIV status (*n *= 35)

	Total	**HIV Negative (***n ***= 11)**	**HIV Positive** **(***n ***= 24)**	*p*-value*
	N (%)	N (%)	N (%)	
**Age,** Med (IQR)	30.0 (25.0–31.0)	27.0 (24.0–35.0)	30.1 (28.0–35.0)	
18–29	16 (45.7)	7 (43.8)	9 (56.2)	0.150
≥ 30	19 (54.3)	4 (21.1)	15 (78.9)	
**Marital status**				
In a relationship/married	7 (20.0)	4 (36.4)	3 (12.5)	0.082
Single, not in a relationship	19 (54.3)	3 (27.3)	16 (66.7)	
Missing	9 (25.7)	4 (36.4)	5 (20.8)	
**Employment**				
Employed	9 (25.7)	2 (18.2)	7 (27.2)	0.442
Unemployed	21 (60.0)	6 (54.5)	15 (62.5)	
Missing	5 (14.3)	3 (27.3)	2 (8.3)	
**Highest education level**				
Less than Grade 12	9 (25.7)	3 (27.3)	6 (25.0)	0.367
Grade 12 and above	8 (22.9)	4 (36.4)	4 (16.7)	
Missing	18 (51.4)	4 (36.4)	14 (58.3)	
**Nationality**				
South African	30 (85.7)	8 (72.7)	22 (91.7)	0.200
Non-South African	2 (5.7)	1 (9.1)	1 (4.2)	
Missing	3 (8.6)	2 (18.2)	1 (4.2)	
**Year of MDR/RR-TB treatment registration**				
2010–2011	8 (22.9)	2 (18.2)	6 (25.0)	0.578
2012–2013	16 (45.7)	4 (36.4)	12 (50.0)	
2014–2016	11 (31.4)	5 (45.5)	6 (25.0)	
**DR-TB treatment site type**				
Centralized site	12 (34.3)	3 (27.3)	9 (37.5)	0.554
Decentralized site	23 (65.7)	8 (72.73)	15 (62.5)	
**Baseline CD4, cells/mm**^**3**^Med (IQR)	-	-	145 (51–301)	-
< 50	-	-	4 (16.7)	-
51–250	-	-	9 (37.5)	-
> 250	-	-	6 (25.0)	-
Missing	-	-	5 (20.8)	-
**ART status**				
On ART	-	-	20 (83.3)	-
ART status unknown	-	-	4 (16.7)	-
**HIV positive on ART before TB treatment**				
Yes	-	-	12 (60.0)	-
No	-	-	6 (30.0)	-
Unknown			2 (10.0)	-
**ART regimen**				
FDC (TDF + 3TC/FTC + EFV/NVP)	-	-	9 (45.0)	-
d4T/AZT-3TC-EFV/NVP	-	-	9 (45.0)	-
d4T-3TC-LPV/r	-	-	1 (5.0)	-
TDF-3TC-LPV/r	-	-	1 (5.0)	-
**Patient category**				
New	19 (54.3)	6 (54.5)	13 (54.2)	0.419
Previously treated	13 (37.1)	3 (27.3)	10 (41.7)	
Unknown	3 (8.6)	2 (18.2)	1 (4.2)	
**Previously treated TB resistance profile**				
RR-TB	2 (15.4)	-	2 (20.0)	1.000
MDR-TB	6 (46.2)	2 (66.7)	4 (40.0)	
Missing	5 (38.5)	1 (33.3)	4 (40.0)	
**Previously treated TB treatment outcomes**				
Completed	6 (46.2)	-	6 (60.0)	0.031
Cured	2 (15.4)	-	2 (20.0)	
Treatment failure	4 (30.8)	3 (100.0)	1 (10.0)	
Unknown	1 (7.7)	-	1 (10.0)	
**TB disease type**				
PTB	22 (62.9)	9 (81.8)	13 (54.2)	0.452
EPTB	9 (25.7)	2 (18.2)	7 (29.2)	
Both PTB and EPTB	3 (8.6)	-	3 (12.5)	
Missing	1 (2.9)	-	1 (4.1)	
**Resistance profile**				
RR-TB	18 (51.4)	2 (18.2)	16 (66.7)	0.009
MDR-TB	12 (34.3)	5 (45.4)	7 (29.2)	
XDR-TB	2 (5.7)	2 (18.2)	-	
Missing	3 (8.6)	2 (18.2)	1 (4.1)	
**TB treatment regimen**				
Standard second-line TB regimen	10 (28.6)	4 (36.4)	6 (25.0)	0.490
Individualized regimen with second-line drugs	25 (71.4)	7 (63.6)	18 (75.0)	
**Pregnant at MDR/RR-TB treatment initiation**				
Yes	31 (88.6)	9 (81.8)	22 (91.7)	0.575
No	4 (11.4)	2 (18.2)	2 (8.3)	
*Time to pregnancy onset, Med (IQR)*	*10.9 (5.5–16.4)*	*5.5 (4.0–7.1)*	*16.4 (14.7–18.1)*	*0.121*

*TB* Tuberculosis, *PTB* Pulmonary TB, *EPTB* Extra-pulmonary TB, *RR-TB* Rifampicin-resistant TB, *MDR-TB* Multidrug-resistant TB, *XDR-TB* Extensively drug-resistant TB, Med median, *IQR* Interquartile range, *ART* Antiretroviral therapy, *FDC* Fixed-dose combination, *TDF* Tenofovir, *EFV* Efavirenz, *NVP* Nevirapine, *d4t* Stavudine, *AZT* Zidovudine, *3TC* Lamivudine, *LPV/r* Lopinavir/ritonavir

*P* values from Chi-square or Fisher’s exact tests. Chi-squared, *P *< 0.05- statistically significant

A total of 24 (68.6%) women were HIV-positive, the median CD4 at ART initiation among women with baseline CD4 counts on file (*n *= 19), was 145 cells/mm3, IQR (51–301). Twenty (83.3%) of the HIV-positive women were initiated on ART with 60% initiated on ART before the start of MDR/RR-TB treatment. While four women had no data regarding ART initiation.

The majority of the women 31/35 (88.6%) were pregnant at the time of MDR/RR-TB treatment initiation. Four women became pregnant at a median time of 10.9 months IQR (5.5–16.4) after MDR/RR-TB treatment initiation, with HIV-negative women becoming pregnant earlier, at a median time of 5.5 months IQR (4.0–7.1).

**Fig. 3:**
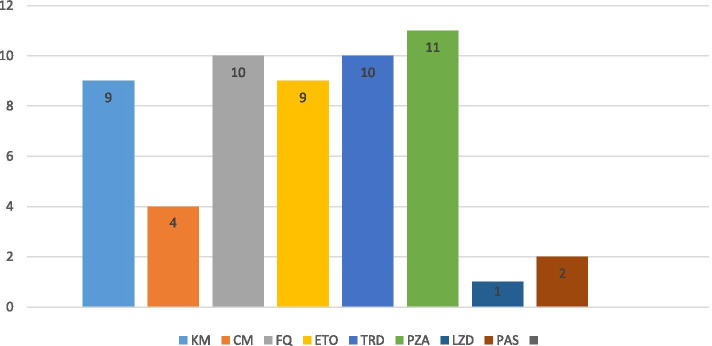
Frequency of anti-TB drugs contained in initial treatment regimens among women pregnant at MDR/RR-TB treatment initiation (*n *= 31). KM – Kanamycin, CM – Capreomycin, FQ – Fluoroquinolones, ETO – Ethambutol, TRD – Terizidone, PZA – Pyrazinamide, LZD – Linezolid, PAS – P-aminosalicyclic acid

More than 50% of the women were resistant to rifampicin only (RR-TB), 34% were resistant to rifampicin and other drugs (MDR-TB), and 2 HIV-negative women had XDR-TB. The majority of second-line anti-TB drug regimens were individualized, with only 28.6% of women receiving standard second-line regimens. Pyrazinamide and terizidone were the most commonly used drugs in the initial treatment regimen in the study cohort (Fig. 3).

**Fig. 4:**
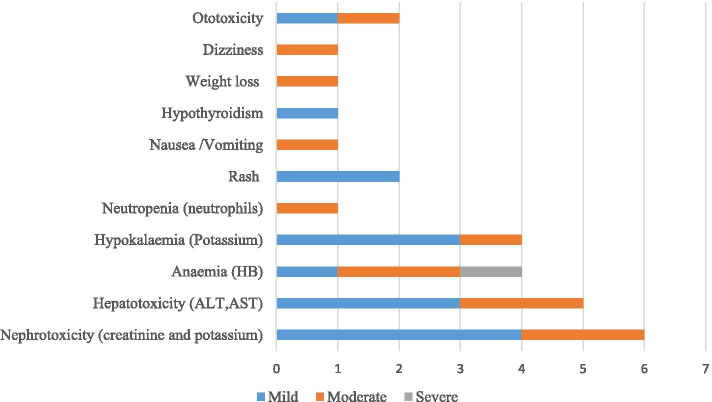
Frequency and severity of adverse drug observed during RR-TB treatment (*n *= 17)

Overall, 17/35 (48.6%) women experienced adverse events during their MDR/RR-TB treatment while also pregnant. The majority, 82.4%, experienced only one adverse event while 17.6% experienced two or more adverse events. Adverse events were more common in HIV-positive (62.5%) vs HIV-negative (18.2%) women. The most prevalent adverse event reported was nephrotoxicity 6/17 (35.3%), of which (2/6) 33.3% were moderate (Fig. 4). This was followed by anaemia 4/17 (23.5%), hypokalaemia 4/17 (23.5%) and equal number 2/17 (11.8%) experiencing ototoxicity or rash. Hypothyroidism, weight loss and dizziness were less common 1/17 (5.9%) respectively.

### Differences in characteristics by HIV status

HIV-negative patients were different from HIV-positive patients in terms of previous TB treatment outcomes, current TB infection profile and occurrence of adverse events (Table 2). A higher proportion of HIV-negative women had a history of TB treatment failure (100% vs 11.1%, *p *< 0.05) and a higher proportion were infected with MDR-TB and XDR-TB, (55.6%, 22.2%, vs 30.4%, 0%, *p *< 0.05), while more HIV-positive patients presented with RR-TB infection (69.6% vs 22.2%, *p *< 0.05). Furthermore, HIV-negative women experienced fewer adverse events (18.2% vs 62.5%; *p *< 0.05).

**Table 2:** Frequency and severity of adverse drugs observed during MDR/RR-TB treatment

	Total (*n *= 35)	**HIV Negative (***n ***= 11)**	**HIV Positive** **(***n ***= 24)**	*p*-value*
	N (%)	N (%)	N (%)	
**Adverse events**				
No adverse events	18 (51.4)	9 (81.8)	9 (37.5)	0.027
Adverse events	17 (48.6)	2 (18.2)	15 (62.5)	
*1 event*	*14 (82.4)*	*2 (18.2)*	*12 (50.0)*	
≥ *2 events*	*3 (17.6)*	*-*	*3 (12.5)*	
**TB regimen change**				
No	14 (40.0)	5 (45.5)	9 (37.5)	0.656
Yes	21 (60.0)	6 (54.5)	15 (62.5)	

*TB* Tuberculosis

*P* values from Chi-square or Fisher’s exact tests. Chi-squared, *P *< 0.05- statistically significant

### Maternal TB treatment and pregnancy outcomes

Seven (20.0%) of the women did not have outcomes assigned because they were transferred out of the study site for continued care. Among women with outcomes assigned, 17/28 (60%**)** completed treatment, of these 4 were cured, whereas 28.6% women were lost to follow-up, and 10.7% died (Table 3).

**Table 3:** Maternal TB and pregnancy outcomes by HIV status (*n *= 35)

	Total *(n *= *28)*	HIV Negative *(n *= *8)*	HIV Positive *(n *= *20)*	*p*-value*
	N (%)	N (%)	N (%)	
TB treatment outcomes				
Completed	13 (46.4)	3 (37.5)	10 (50.0)	0.759
Cured	4 (14.3)	2 (25.0)	2 (10.0)	
Treatment failure	-	-	-	
LTFU	8 (28.6)	2 (25.0)	6 (30.0)	
Died	3 (10.7)	1 (12.5)	2 (10.0)	
	Total *(n* = *35)*	HIV Negative *(n* = *11)*	HIV positive *(n* = *24)*	*p*-value
Pregnancy outcomes	N (%)	N (%)	N (%)	
Unknown pregnancy outcomes	15 (43.0)	6 (40.0)	9 (60.0)	
Known pregnancy outcomes	20 (57.0)	5 (25.0))	15 (75.0)	
Live birth (full term)	4 (20.0)	2 (40.0)	2 (13.3)	0.144
Preterm birth	11 (55.0)	1 (20.0)	10 (66.7)	
Miscarriage	1 (5.0)	-	1 (6.7)	
Neonatal death	1 (5.0)	-	1 (6.7)	
Termination	3 (15.0)	2 (40.0)	1 (6.6)	

*TB* Tuberculosis, *LTFU* Lost to follow up

*P* values from Chi-square or Fisher’s exact tests. Chi-squared, *P *< 0.05- statistically significant

Pregnancy outcomes were available for 20/35 (57.1%) women. There were 15 live births documented (11 preterm), one miscarriage, one neonatal death and three pregnancy terminations. Overall, 13/20 (65.0%) of the women with known pregnancy outcomes had an adverse pregnancy outcome. TB treatment and pregnancy outcomes did not differ by HIV status among those with known outcomes.

## Discussion

In this cohort of 35 pregnant women receiving second-line anti-TB treatment for MDR/RR-TB in Johannesburg, South Africa, fifty-seven percent had known pregnancy outcomes. Of these, almost two-thirds experienced adverse pregnancy outcomes, which, excluding three elective pregnancy terminations, included preterm birth (55%), neonatal death (5%) and miscarriage (5%). The reported preterm births in our cohort were higher than was previously reported in a cohort of 108 pregnant women managed for MDR/RR-TB in KwaZulu-Natal in South Africa between 2013 and 2017 (28%), and a cohort of 38 pregnant women treated for MDR/RR-TB in Lima, Peru between 1996 and 2005 (2.7%) [13, 22]. Most concerning is that the preterm births in our cohort were also higher than in HIV and general populations in South Africa which range from 8% to over 30% [23–26]. Multiple factors may explain the observed differences in pregnancy outcomes compared to previous cohorts. Firstly, there is an increased risk poor maternal and birth outcomes pregnant women with HIV and TB co-infection as compared to women with HIV who do not have TB [27, 28]. Almost all of the adverse birth outcomes, including preterm births, were among HIV-positive women in our cohort. This is not unexpected considering that two-thirds of the women living with HIV. Secondly, although not definitive, ART use during pregnancy has been shown in some studies to be associated with increased risk of preterm birth compared to preterm birth rates among HIV negative women [24, 29]. Although a higher proportion of the KwaZulu-Natal cohort were HIV infected (81%), our cohort had more severe immunosuppression, presenting with a median baseline CD4 counts of 145cells/mm^3^, compared to the baseline CD4 count of 343 cells/mm^3^ among the KwaZulu-Natal cohort. The Peru cohort only had 8% of women co-infected with HIV which may explain the higher proportion of favourable pregnancy outcomes compared to both the KwaZulu-Natal cohort and the current study. These findings suggest that RR/MDR-TB and HIV co-infection during pregnancy are a cause of poor birth outcomes highlighting the need for intensified TB prevention strategies, and adequate TB treatment in antenatal care and HIV programs.

Overall, 60.1% of the women in our study successfully completed MDR/RR-TB treatment, with 11.4% cured. These outcomes are comparable to the national drug-resistant TB success of 54% for South Africa in 2016 and similar to the treatment success rate in previous studies of pregnant women with RR/MDR-TB. The KwaZulu-Natal cohort had slightly better TB outcomes of 67% treatment success rate [22, 30]. However, half of this cohort received bedaquiline as part of their TB treatment regimen, which has shown improved treatment success rates among MDR/ RR-TB patients [13].

The majority of the women in our study were pregnant at the time of MDR/RR-TB diagnosis. Those that became pregnant after TB treatment initiation became pregnant during the continuation phase of treatment when most patients start to feel better and start resuming normal life activities. The lack of contraceptive use in these patients is a cause for concern as it is specified as part of the management of MDR/RR-TB in women of childbearing age [9]. Because of the lack of safety data in pregnancy for many second-line TB drugs, and the potential for poor TB and maternal outcomes, the South African National department of health guidelines recommend that all women of child-bearing potential with MDR/RR-TB should be offered reproductive counselling and access to family planning [9].

The strength of our study is that it is the first to describe the occurrence of specific drug-related adverse events among women treated with second-line TB drugs while pregnant. Almost 48.5% of the women experienced at least one adverse event, which is lower than 83% that was reported previously in a systematic review to estimate the prevalence of adverse events during DR-TB treatment among non-pregnant patients [31]. Higher rates of adverse events occurred among HIV-positive mothers, likely due to the concurrent use of ART [31]. Hepatotoxicity and nephrotoxicity were most prevalent; they have been linked to pyrazinamide, ethionamide, kanamycin and fluoroquinolones [32, 33] which were the most commonly used drugs to treat our study cohort. Aminoglycosides and ethionamide have since been removed from the new all-oral short and long course MDR/RR regimens in South Africa, but given the increasing availability of new drugs such as bedaquiline and linezolid their use among pregnant women should be reconsidered for countries still using the long course regimens for MDR/RR-TB [2].

### Limitations

The retrospective design might have introduced selection or information bias. Women were referred to other facilities for ANC and delivery; therefore, birth and pregnancy outcomes were poorly documented in the TB records reviewed. Reporting of adverse events was incomplete, and some laboratory result forms were missing from patient files, likely resulting in an underestimation of adverse events. Additionally, since our data relies on the accurate reporting of pregnancy in the medical record, *n *= 35 may be an underestimate if not all the pregnancies were recorded.

Although the data was collected from 3 main hospitals, the sample size was very small; therefore, findings may not be generalizable and applicable to other populations.

## Conclusions

Pregnant women with MDR-TB/RR-TB suffer from high rates of adverse pregnancy outcomes. Results from our study highlight the need for close monitoring and coordinated obstetric, HIV and TB care for these vulnerable patients.

## Data Availability

The datasets generated and/or analyzed during the current study are not publicly available as the data are owned by the study sites and the National Department of Health (South Africa) and governed by the Human Research Ethics Committee (University of the Witwatersrand, Johannesburg, South Africa). All relevant data are included in the paper. The full data are available from the Health Economics and Epidemiology Research Office for researchers who meet the criteria for access to confidential data and with permission from the owners of the data. Contact the organization at information@heroza.org for additional information regarding data access.

## Abbreviations

ANC: Antenatal care
ART: Antiretroviral therapy
CD4: Cluster of differentiation 4
DAIDS: Division of acquired immunodeficiency syndrome
DR-TB: Drug-resistant tuberculosis
HIV: Human immunodeficiency virus
HREC: Human Research Ethics Committee
INH: Isoniazid
IPT: Isoniazid preventative therapy
IQR: Interquartile range
LTFU: Lost to follow-up
MDR/RR-TB: Multi-drug resistant and rifampicin-resistant tuberculosis
NHLS: National Health Laboratory Services
RIF: Rifampicin
TB: Tuberculosis
WHO: World Health Organisation
XDR-TB: Extensively drug-resistant tuberculosis
